# Prediction of Femtosecond Laser Etching Parameters Based on a Backpropagation Neural Network with Grey Wolf Optimization Algorithm

**DOI:** 10.3390/mi15080964

**Published:** 2024-07-28

**Authors:** Yuhui Liu, Duansen Shangguan, Liping Chen, Chang Su, Jing Liu

**Affiliations:** 1 School of Mechanical Science and Engineering, Huazhong University of Science and Technology, Wuhan 430074, China; yuhuiliu@hust.edu.cn (Y.L.); ahcq1990@hust.edu.cn (D.S.); chenlp@hust.edu.cn (L.C.); 2 College of Computer Science, South-Central Minzu University, Wuhan 430074, China

**Keywords:** laser etching, prediction, backpropagation neural network, grey wolf optimization, model evaluation

## Abstract

Investigating the optimal laser processing parameters for industrial purposes can be time-consuming. Moreover, an exact analytic model for this purpose has not yet been developed due to the complex mechanisms of laser processing. The main goal of this study was the development of a backpropagation neural network (BPNN) with a grey wolf optimization (GWO) algorithm for the quick and accurate prediction of multi-input laser etching parameters (energy, scanning velocity, and number of exposures) and multioutput surface characteristics (depth and width), as well as to assist engineers by reducing the time and energy require for the optimization process. The Keras application programming interface (API) Python library was used to develop a GWO-BPNN model for predictions of laser etching parameters. The experimental data were obtained by adopting a 30 W laser source. The GWO-BPNN model was trained and validated on experimental data including the laser processing parameters and the etching characterization results. The R^2^ score, mean absolute error (MAE), and mean squared error (MSE) were examined to evaluate the prediction precision of the model. The results showed that the GWO-BPNN model exhibited excellent accuracy in predicting all properties, with an R^2^ value higher than 0.90.

## 1. Introduction

With the rapid development of high-precision and high-efficiency manufacturing, femtosecond laser etching, having an ultrashort duration and an ultrahigh peak power [1], has shown great potential for use in the microelectronic device packaging, microfluid channel manufacturing, aerospace, and biomedical fields [2,3,4,5]. Currently, the planning and optimization of laser processing parameters must be conducted through a cumbersome and extensive trial-and-error basis while considering a large parameter space, including parameters such as pulse energy, frequency, repetition rate, scanning velocity, and number of exposures. The optimization process takes considerable time and energy for each material and each application [6]. Laser processing involves a series of complex physical processes such as multiphoton absorption and avalanche ionization, and is affected by the temperature of the electrons and the lattice [7]. Scaling these physical mechanisms to simulate spatial profiles after multiple-pulse irradiation is still a significant challenge. Many authors have tried to estimate the depth and roughness of a machined surface using empirical models based on energetic considerations [8], to fit the groove profile with a Gaussian function [9], or to model the laser processes behaviors based on finite element analysis [10,11] or Comsol [12]. However, each method shows limitations that relegate their implementation to a specific case or to a limited number of applications.

Recent advances in artificial intelligence, machine learning, and deep learning have given scientists great opportunities to predict the laser etching parameters in the trial-and-error process [13,14,15]. These methods have been used in a wide range of laser machining tasks such as in the real-time monitoring of beam aberrations [16] and preventing overmachining [17] to predicting the hardness distribution of heat-treated steel [18]. The convolutional neural network (CNN) framework has been used to predict the contact angle evolution curves of laser-textured surfaces with diverse micro/nanostructures [19,20,21]. As a type of “machine learning,” the backpropagation neural network (BPNN) [22,23], in particular, has seen increased usage for information processing due to its self-organization, self-learning, knowledge reasoning self-adaptation for nondeterministic regular parameters, and other characteristics. The main features of BPNNs are signal forward propagation and error backpropagation. If the output signal does not meet the expected output requirements, it is transferred to the error backpropagation process, and the weights and thresholds of the network are continuously corrected from the output layer to the input layer according to the prediction error so that the prediction output of the BP neural network continues to approach the expected output. But, it has two drawbacks. One is that it is prone to becoming stuck in local minima, and the other is that the convergence speed is slow.

Nowadays, heuristic algorithms have been proposed, demonstrating immense potential and advantages in solving complex optimization problems. They are capable of providing a feasible solution to a problem within acceptable computational costs (in terms of time and space), and the deviation of this feasible solution from the optimal solution can typically be controlled within a certain range. Examples of such algorithms include genetic algorithms (GAs) [24], particle swarm optimization (PSO) [25], ant colony optimization (ACO) [26], grey wolf optimization (GWO) [27,28], and a multiobjective slime mold algorithm (MOSMA) [29]. GAs [24] have a strong search capability and are suitable for large-scale and nonlinear problems, but they are sensitive to parameter selection. PSO [25] possesses numerous advantages in laser processing prediction; it also exhibits drawbacks such as being prone to becoming trapped in local optima, issues with convergence accuracy, and limited capability in handling discrete and combinatorial optimization problems. Ant colony optimization (ACO) [26], when subjected to improper parameter settings, can suffer from excessively slow convergence speed, so is unsuitable for real-time prediction sand rapid responses to processing demands. The GWO algorithm is a bio-inspired method, which was developed by Mirjalili et al. [30]. The GWO algorithm exhibits outstanding performance in terms of solution accuracy and stability, while also demonstrating low sensitivity to the initial population and resistance to becoming trapped in local optima. Tian [31] and Akanksha [32] have applied the adaptive discrete GWO and response surface GWO to optimize the laser processing path and to reduce the heat-affected zone in laser cutting.

As shown by the previous results and relevant references, the GWO algorithm appears to be a promising meta-heuristic technique for solving different standard optimization problems, which mimics the social hierarchy and hunting capability of grey wolves. In this study, we utilized the GWO algorithm to optimize the hyperparameters (epoch and neuron number) for training a BPNN. The GWO-BPNN model exhibited a significantly enhanced capability to predict the depth, width, and aspect ratio of microgrooves, outperforming the BPNN model. Additionally, the R^2^ score, mean absolute error (MAE), and mean squared error (MSE) were examined to verify the accuracy of GWO-BPNN.

## 2. Equipment, Materials, Experimental Procedures, and Data

### 2.1. Equipment and Materials

**Figure 1:**
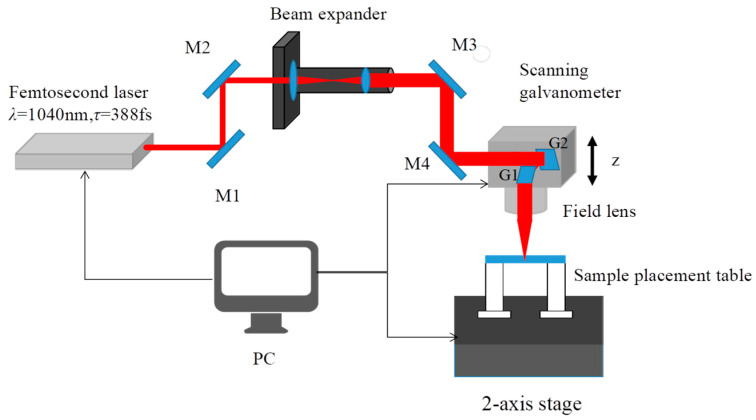
Experimental setup schematic for laser drilling of microgrooves on quartz glass surface.

The experiments were performed on a laser processing system scanning galvanometer with a femtosecond laser (Newport Corporation SPIRIT 16-HE-SHG (Irvine, CA, USA), wavelength λ = 1040 nm, pulse duration *τ* = 388 fs, pulse repetition rate *f* = 100 kHz). The platform was driven by a 5-axis system (X, Y, Z axes and x, y axes); the X and Y axes consisted of a two-axes stage with a 200 mm travel range, 1 µm minimum displacement, and 1.5 µm repeat positioning accuracy, which we used to move the sample arbitrarily in the X and Y axes. The Z axis was driven by a vertical Z-orientation positioner (with 1 µm resolution) to control the vertical position of the laser focus. Along axes x and y, we moved the G1 and G2 mirrors quickly using a scanning galvanometer (Scanlab IntelliSCAN 10 (Puchheim, Germany), 15 mm numerical aperture, 72 mm scanning displacement, 1.2 ms reaction time, and 6 m/s maximum scanning speed), to control the etching path of laser beam on the materials. The collimated near-infrared laser beam passed through mirrors M1 and M2, and then entered the beam expander (beam expansion ratio 1:4). After that, the laser beam was focused onto the scanning galvanometer through mirrors M3 and M4. Finally, it was focused on the sample surface through a field lens (*f* = 100 mm). We investigated the femtosecond-laser-etched microgrooves with diverse depth-to-width ratios on the surface of quartz glass etched with a single-line etching method. The experimental material was JGS1 quartz glass wafer, with a diameter of 25 mm and a thickness of 0.25 mm. Before the experiment, the sample surface was wiped with hydrated alcohol to remove impurities to ensure the sample surface was clean. A schematic of the experimental setup is shown in Figure 1.

### 2.2. Experimental Procedures

**Figure 2:**
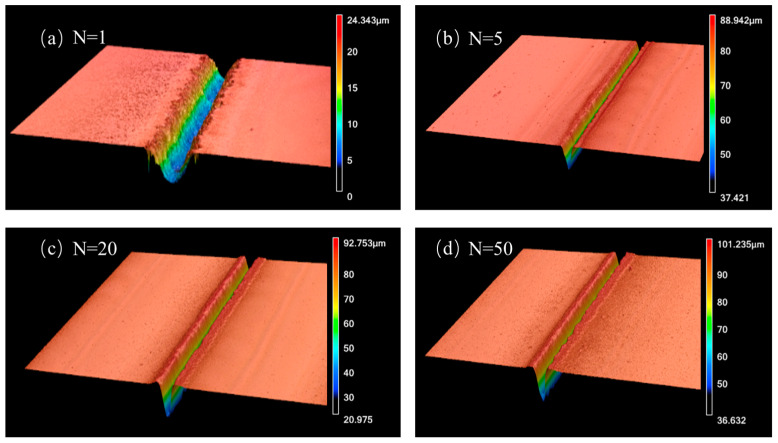
Three-dimensional images of microgrooves obtained with single-pulse laser energy *E_p_* = 40 μJ and scanning velocity *v* = 100 mm/s with the following number of exposures: (**a**) *N* = 1, (**b**) *N* = 5, (**c**) *N* = 20, (**d**) *N* = 50.

The microgroove etching tests were executed on quartz glass, 25 × 25 mm, in the plane dimensions. The laser scanning method was single-line etching, with a length of 1 mm. To research the etching effects for different variables, we adopted a single-variable method and studied the depth, width, and ratio of microgrooves for different single pulse laser energies *E_p_*, different scanning velocities *v*, and different numbers of exposures *N*. The 3D topographic images of the microgrooves obtained with different numbers of exposures *N* = 1, 5, 20, and 50, when laser energy *E_p_* = 40 μJ and scanning velocity *v* = 100 mm/s are displayed in Figure 2. The three-dimensional (3D) topographic images were captured and studied using a laser confocal microscope (Keyence VK-X2500, Osaka, Japan) [33]. With this 3D confocal microscope, the sample information was collected with an objective lens under the illumination of a light-emitting diode (LED) point light source and focused on the detector via a beam splitter. When the laser scanned the sample point by point, the photomultiplier tube behind the detector captured the image point by point and converted it into a digital signal, which was then transmitted to a computer to form a confocal image using software. The maximum observation magnification of the confocal microscope was 24,000 times, the image resolution was 0.13 μm, the displacement accuracy of the horizontal moving platform in the x and y axes was 1 μm, and the maximum accuracy in the z-axis direction was 1 nm.

### 2.3. Data Collection

**Figure 3:**
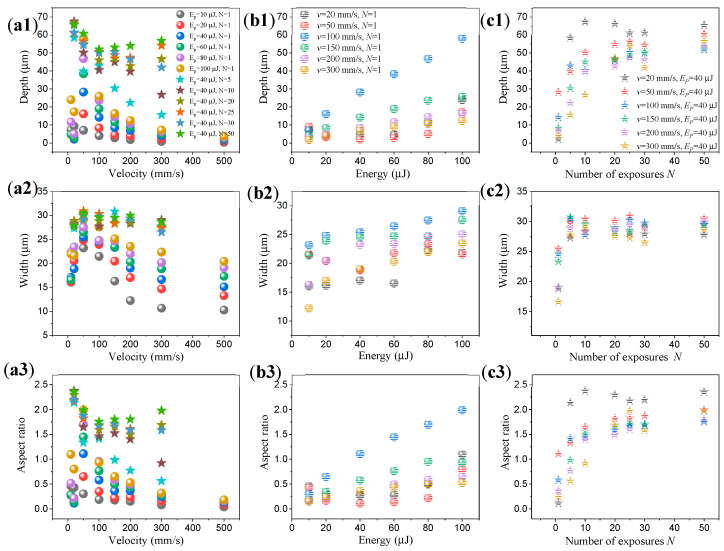
The interdependence of the three physical properties with the characterization (depth, width and aspect ratio) of the laser etching results: (**a1**~**a3**) share a legend with (**a1**); (**b1**~**b3**) share a legend with (**b1**); (**c1**~**c3**) share a legend with (**c1**).

While performing the laser etching process, the single pulse laser energy E_p_, different scanning velocity *v*, and number of exposures *N* were given as the inputs X to the deep learning program. The surface characteristics of the microgrooves were evaluated as width, depth, and aspect ratio as the outputs Y of the deep learning program. These data constituted the three inputs and three outputs. A total of 96 sets of data were used for training, and 24 sets of data were used for validation. All the data are shown in Figure 3, whereas we can find that a single parameter does not provide any determining information about the laser etching formation. Notably, the aspect ratio value is not a new formula: it is the division of the width by the depth; yet, it provides a parametric approach for depth–width prediction.

**Figure 4:**
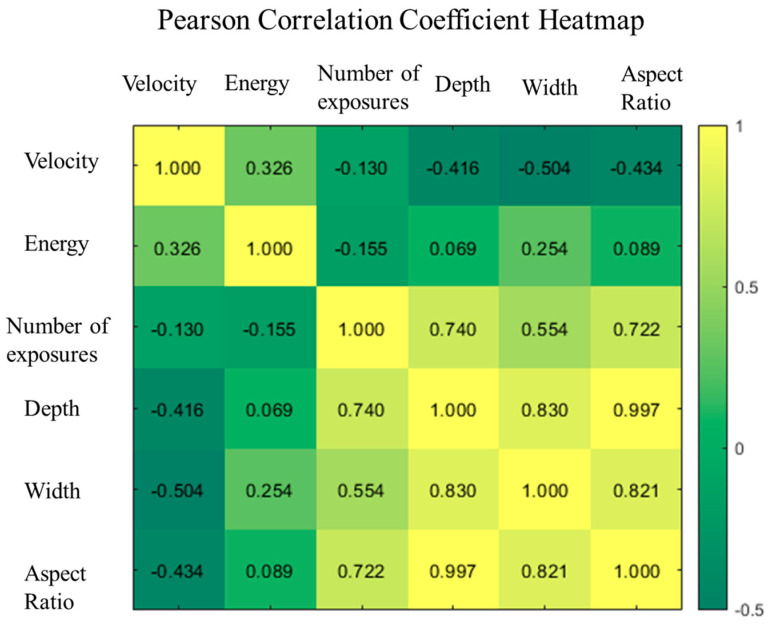
Pearson correlation matrix between any two features.

Measuring the dependence between random observations plays a central role in statistics. Since it is very difficult to fully understand and describe dependencies, one is often interested in condensing the strength of the dependence into one single number. The classical and arguably most popular correlation coefficient is the Pearson coefficient, which, for random variables *X* and *Y* with finite and positive variances, is defined as [34]

(1) $$cor(X,Y)=\frac{\mathrm{cov}(X,Y)}{\sqrt{Var(X)Var(Y)}}$$

where cov(*X, Y*) denotes the covariance of *X* and *Y*. These six parametric approaches were analyzed statistically. The Pearson correlation test coefficients of each parameter are shown in the heat map in Figure 4. As a result, there was no strong correlation (*P* ~ 1.0) between any of these features. In this experiment, it was found that the Pearson correlation coefficients among the features were relatively low. So, all of them were retained since each feature contributed individually to the model. Simultaneously, the output exhibited a very weak linear connection with the input variables. 

## 3. GWO-BPNN Architecture

### 3.1. GWO-BPNN Model Structure

**Figure 5:**
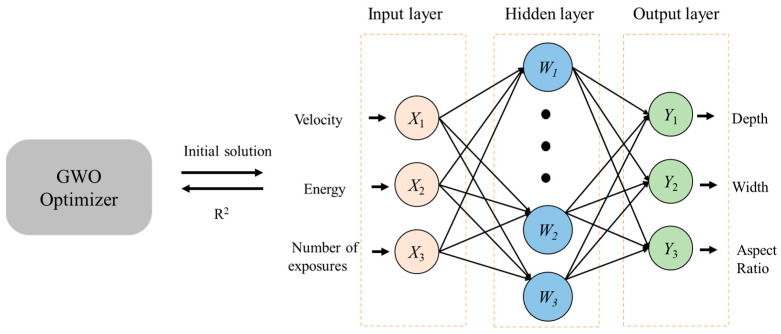
Basic structure of the GWO-BPNN model.

The Keras application programming interface (API) Python library was used to develop the GWO-BPNN model for predictions of laser etching parameters. The BPNN consists of three layers: an input layer, a hidden layer, and an output layer, which is shown in Figure 5. Notably, the hidden layer can have multiple layers. X_1_, X_2_, …, X_n_ represent the input values of the BPNN. W_1_, W_2_, …, W_p_ represent the hidden layer nodes; and Y_1_, Y_2_, …, Y_m_ represent the output values. It is worth noting that dropout was applied during GWO-BPNN training to reduce the model’s dependence on the initial data and improve its generalization ability.

In the process of network training, it is hard to determine the number of neurons in the hidden layer. If the number of neurons is small, the network has a weak ability to recognize samples. If the number of neurons is large, the number of network iterations increases, which prolongs the training time of the network; at the same time, the generalization ability of the network is reduced, resulting in the decline in the prediction ability of the network. However, there is no method that is more accurate for determining the number of neurons in the hidden layer, and the number of neurons in the hidden layer can only be determined using empirical formulas and multiple experimental comparisons.

In this study, the number of neurons in the hidden layer and epochs were optimized using GWO to improve the performance of the BPNN. The GWO algorithm imitates the behavior of grey wolves to reach the prey’s position. It contains four types of wolves in a group, namely α, β, δ, and ω. The top level of the hierarchy has α as the group leader. ω wolves are the lowest-ranking wolves. The hunting processes include encircling, hunting, and attacking prey. The following describes how gray wolves’ behavior toward nearby prey is mathematically modeled [27,35]:

(2) $$D_{i}=\left|W_{prey}\cdot X_{prey}-X_{i}\right|$$

(3) $$X_{i}=X_{prey}-A_{i}\cdot D_{i}$$

where $X_{prey}$ is the position of the prey, $X_{i}$ is the position vector of the grey wolf, and $A_{i}=2\cdot rand\cdot a\text{-}a$ and $W_{prey}=2\cdot rand$ are coefficient vectors. Rand represents random vectors.

After the gray wolves surround the prey, they hunt for it. The first three best answers are kept, and the other wolves’ position are updated, was be illustrated by [35]

(4) $$\begin{array}{l} D_{\alpha }=\left|W_{\alpha }\cdot X_{\alpha }-X_{i}\right|\\ D_{\beta }=\left|W_{\beta }\cdot X_{\beta }-X_{i}\right|\\ D_{\delta }=\left|W_{\delta }\cdot X_{\delta }-X_{i}\right| \end{array}$$

where $W_{\alpha }$,$W_{\beta }$,$W_{\delta }$ can be expressed by 2⋅rand.

Here, the three best wolves are constantly updated in each iteration,

(5) $$\begin{array}{l} X_{i\alpha }=\left|X_{\alpha }-A_{\alpha }\cdot D_{\alpha }\right|\\ X_{i\beta }=\left|X_{\beta }-A_{\beta }\cdot D_{\beta }\right|\\ X_{i\delta }=\left|X_{\delta }-A_{\delta }\cdot D_{\delta }\right| \end{array}$$

(6) $$\mathrm{and}\;X_{i+1}=\frac{X_{i\alpha }+X_{i\beta }+X_{i\delta }}{3}$$

where $X_{i+1}$ represents the prey’s new position.

R^2^ was used as a fitness function to demonstrate the performance of the GWO in the training phase, which is calculated as

(7) $$R^{2}=1-\frac{\sum_{i=1}^{n}{\left(Y_{real,i}-Y_{pre,i}\right)}^{2}}{\sum_{i=1}^{n}{\left(Y_{real,i}-\bar{Y}\right)}^{2}}$$

where $Y_{real,i}$ is the actual value, $Y_{pre,i}$ is the predicted value, $\bar{Y}$ is the mean value, and *n* is the total number of data points.

In addition, the numbers of neurons in the hidden layer and epochs were changed and adjusted to minimize the fitness function of training. The hunting process of GWO is carried out until the stop criterion is met or the set number of iterations is achieved.

### 3.2. Performance Evaluation

The regression performance of GWO-BPNN was determined using different evaluation metrics. The most commonly used metrics for this purpose are the mean absolute error (MAE), R^2^, and mean square error (MSE). The MAE and MSE can be calculated as

(8) $$MAE=\frac{1}{n}\sum_{i=1}^{n}\left(Y_{real,i}-Y_{pre,i}\right)$$

(9) $$MSE=\frac{1}{n}\sum_{i=1}^{n}{\left(Y_{real,i}-Y_{pre,i}\right)}^{2}$$

## 4. Results and Discussion

**Figure 6:**
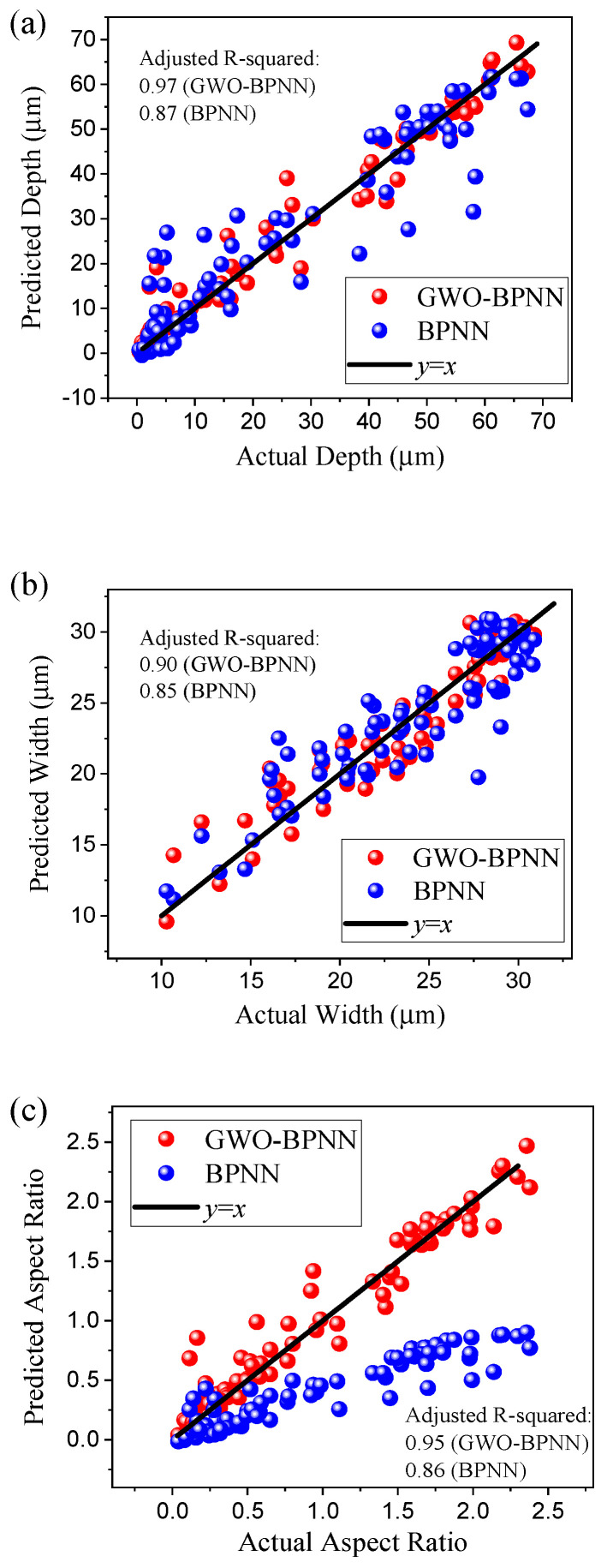
Comparison of GWO-BPNN-predicted values, BPNN-predicted values, and experimental data. (**a**) Depth, (**b**) width, (**c**) aspect ratio of microgrooves.

Figure 6 presents a comparison between GWO-BPNN- and BPNN-predicted values and the experimental data on the training sets and testing sets. It can be seen that with GWO-BPNN, all the data points are densely distributed along the black line of y = x, implying the equivalence between the predicted values and the actual data. In comparison, the predicted values of BPNN are quite dispersed, leading to a substantial deviation between the predicted aspect ratio values and the actual aspect values. In the predicted depth, the adjusted R-squared of GWO-BPNN was 0.97, which is higher than the 0.87 of BPNN. Similarly, the adjusted R-squared of GWO-BPNN for the predicted width and aspect ratio were 0.90 and 0.95, but 0.85 and 0.86 for BPNN, respectively. The closer the adjusted R-squared value is to one, the better the model fit. Therefore, compared to BPNN, the GWO-BPNN model demonstrated a superior ability to perfectly predict the depth, width, and aspect ratio of the microgrooves. Additionally, no outliner was observed for any of the features.

To demonstrate the role of GWO, the MAE, R^2^ and MSE for the predictions using the GWO-BPNN and BPNN models are shown in Table 1. The R^2^ values of the GWO-BPNN model were larger than 0.9, implying excellent predictive ability. When comparing the two models, the R^2^ of the aspect ratio for GWO-BPNN was 0.907, while that of BPNN was 0.782. Notably, the R^2^ differences in depth and width were relatively small between GWO-BPNN and BPNN. This indicated that GWO significantly improved the stability and prediction accuracy of the BPNN model. In addition, the MAE and MSE values of all the features were fairly acceptable.

**Figure 7:**
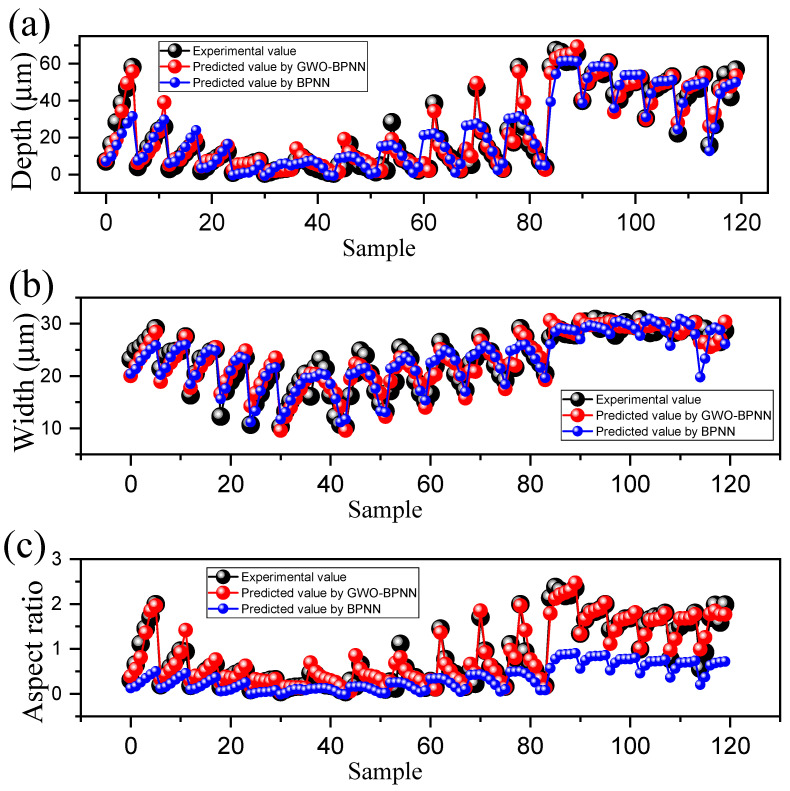
Comparison between the actual with GWO-BPNN- and BPNN-predicted output variables. (**a**) Depth, (**b**) width, (**c**) aspect ratio of the microgrooves.

During the training process, while the predicted values for depth and width were individually accurate, when they were combined into the aspect ratio, small numerical differences were amplified, leading to a decline in the overall prediction performance. Therefore, we needed to impose a constraint condition when predicting the results, to ensure that the depth, width, and aspect ratio were greater than 0 to guarantee that the data possessed physical significance and to improve prediction accuracy and reliability. Figure 7 illustrates the output variables obtained from GWO-BPNN and BPNN prediction and their comparison with the actual values. Compared with those of BPNN-predicted data, the curve behaviors of GWO-BPNN-predicted data were sufficiently consistent with the actual values, meaning that GWO was efficient in improving the prediction ability of the BPNN model. Notably, the aspect ratios of the microgroove predicted by BPNN were significantly different from the actual values, as shown in Figure 7c. The BPNN adopts the gradient descent method for weight adjustment, and the objective function is usually quite complex, which leads to the “zigzag phenomenon” during the training process. That is, the weights fluctuate near the optimal value, making it easy for the algorithm to converge to a local minimum rather than the global optimal solution. By integrating GWO with BPNN, the GWO-BPNN model can overcome some of the challenges associated with traditional neural network training methods, such as sensitivity to initial weights and local minima issues, thus enhancing the overall performance and accuracy of the neural network in solving complex problems.

## 5. Conclusions

In conclusion, an innovative model, BPNN hybridized with GWO, was developed to predict multiple properties of microgrooves: depth, width, and aspect ratio. Compared with the original BPNN model, the GWO-BPNN model exhibited better prediction performance for all the targets, with R^2^ values higher than 0.9, indicating that the GWO significantly improved the prediction ability of the BPNN model. In addition, the GWO-BPNN model learns with examples: it does not require deep knowledge of the process mechanisms or specific mathematical relationship. Moreover, GWO-BPNN also has strong adaptability and generalization ability to adapt to the various demands regarding different materials and processing conditions. It is worth mentioning that the GWO-BPNN model optimizes the parameters of the backpropagation neural network (BPNN) through the grey wolf optimizer (GWO) algorithm, overcoming the sensitivity of the BPNN to the initial parameters. In the future, more input parameters can be considered, such as laser parameters (pulse duration, wavelength, beam profile, etc.), material properties (bandgap, dielectric coefficient, damage threshold, etc.), and laser processing parameters (velocity, energy, number of exposures, etc.) in order to strengthen the generalization capabilities of the model. Meanwhile, various technical means, such as data preprocessing, regularization, and cross-validation, can be used to improve the model’s overall accuracy and reliability. These technical means are highly result-oriented, smarter, useful, and less expensive than conventional techniques. In the future, we plan to investigate other metaheuristics-based BPNN architectures to compare the effectiveness, the computational cost, and complexity with those of the proposed model.

## Figures and Tables

**Table 1:** Evaluation parameters of the GWO-BPNN and BPNN models.

	GWO-BPNN			BPNN		
	MAE	R^2^	MSE	MAE	R^2^	MSE
**Depth**	1.127	0.968	3.390	1.235	0.970	2.728
**Width**	1.610	0.947	5.212	2.606	0.907	9.344
**Aspect ratio**	2.525	0.907	14.32	4.110	0.782	32.48

## Data Availability

All data generated and analyzed during the current study are available from the corresponding author upon reasonable request.
